# New microRNA Biomarkers for Drug-Induced Steatosis and Their Potential to Predict the Contribution of Drugs to Non-alcoholic Fatty Liver Disease

**DOI:** 10.3389/fphar.2017.00003

**Published:** 2017-01-25

**Authors:** Mireia López-Riera, Isabel Conde, Laia Tolosa, Ángela Zaragoza, José V. Castell, María J. Gómez-Lechón, Ramiro Jover

**Affiliations:** ^1^Unidad de Hepatología Experimental, Instituto de Investigación Sanitaria La Fe, Hospital Universitari i Politècnic La FeValencia, Spain; ^2^Servicio Medicina Digestiva, Sección Hepatología, Hospital Universitari i Politècnic La FeValencia, Spain; ^3^Centro de Investigación Biomédica en Red de Enfermedades Hepáticas y Digestivas (CIBERehd), Instituto de Salud Carlos IIIMadrid, Spain; ^4^Departamento de Bioquímica y Biología Molecular, Facultad de Medicina, Universitat de ValènciaValencia, Spain

**Keywords:** drug-induced steatosis, predictive biomarker, microRNA, metabolic syndrome drug, hepatosteatosis, non-alcoholic fatty liver disease

## Abstract

**Background and Aims:** Drug-induced steatosis is a major reason for drug failure in clinical trials and post-marketing withdrawal; and therefore, predictive biomarkers are essential. These could be particularly relevant in non-alcoholic fatty liver disease (NAFLD), where most patients show features of the metabolic syndrome and are prescribed with combined chronic therapies, which can contribute to fatty liver. However, specific biomarkers to assess the contribution of drugs to NAFLD are lacking. We aimed to find microRNAs (miRNAs) responsive to steatotic drugs and to investigate if they could become circulating biomarkers for drug-induced steatosis.

**Methods:** Human HepG2 cells were treated with drugs and changes in miRNA levels were measured by microarray and qRT-PCR. Drug-induced fat accumulation in HepG2 was analyzed by high-content screening and enzymatic methods. miRNA biomarkers were also analyzed in the sera of 44 biopsy-proven NAFLD patients and in 10 controls.

**Results:** We found a set of 10 miRNAs [miR-22-5p, -3929, -24-2-5p, -663a, -29a-3p, -21 (5p and 3p), -27a-5p, -1260 and -202-3p] that were induced in human HepG2 cells and secreted to the culture medium upon incubation with model steatotic drugs (valproate, doxycycline, cyclosporin A and tamoxifen). Moreover, cell exposure to 17 common drugs for NAFLD patients showed that some of them (e.g., irbesartan, fenofibrate, and omeprazole) also induced these miRNAs and increased intracellular triglycerides, particularly in combinations. Finally, we found that most of these miRNAs (60%) were detected in human serum, and that NAFLD patients under fibrates showed both induction of these miRNAs and a more severe steatosis grade.

**Conclusion:** Steatotic drugs induce a common set of hepatic miRNAs that could be used in drug screening during preclinical development. Moreover, most of these miRNAs are serum circulating biomarkers that could become useful in the diagnosis of iatrogenic steatosis.

## Introduction

Drug-induced liver injury is the major reason for drug failure in clinical trials and for the withdrawal of commercialized drugs (Giacomini et al., 2007). Moreover, DILI is a primary cause of acute liver failure, as it makes up to half of all acute liver failures in the clinic (Larrey and Pageaux, 2005). DILI can arise by diverse mechanisms and result in various clinical manifestations such as hepatocellular lesions, necrosis, fibrosis, cholestasis, steatosis, and phospholipidosis. Steatosis is triggered by a plethora of drugs in current use and refers to the impairment of normal synthesis and elimination of TG, leading to an abnormal retention of lipids within hepatocytes (Amacher and Chalasani, 2014). Among the drugs causing steatosis we found tetracyclines, VALP, AMIO, TAMO and CYCA (Donato and Gomez-Lechon, 2012; Amacher and Chalasani, 2014).

Drug-induced steatosis has the potential to cause or contribute to NAFLD, a leading cause of liver disease in developed countries, where it affects one-third of adults and an increasing number of children. The disease begins with an abnormal accumulation of TG in the liver, which in some individuals triggers NASH, characterized by hepatocellular injury, inflammation, and even fibrosis. NASH, in turn, can progress to cirrhosis and hepatocellular carcinoma (Farrell and Larter, 2006; Cohen et al., 2011). It is estimated that only a small number of NASH cases (∼2%) are directly induced by marketed drugs (Grieco et al., 2005). However, many other drugs may exacerbate or precipitate fatty liver or NASH in the presence of other risk factors (Ramachandran and Kakar, 2009). This could be particularly relevant in NAFLD patients with MS-associated risk factors, which are exposed to a large daily number of drugs for hypertension, diabetes, dyslipidemias, etc. However, the potential contribution of these drugs to fatty liver or NAFLD progression has never been investigated.

Preclinical detection of adverse-drug reactions has failed to detect about 40% of potentially hepatotoxic compounds in humans (Peters, 2005). Therefore, the development of new biomarker strategies and approaches is essential. Recently, circulating miRNAs have been studied as emerging biomarkers that promise early response and increased sensitivity (Amacher et al., 2013). These features are particularly relevant for DILI as conventional biomarkers, such as the serum transaminases and bilirubin, are induced only after significant liver damage has already occurred. Acetaminophen overdose, for instance, usually produces no symptoms during the first 24 h after ingestion, followed by the onset of liver failure. However, in this context, some miRNAs (miR-122 and miR-192) showed an earlier and less variable elevation in plasma than ALT (Wang et al., 2009), which suggested that circulating miRNAs may be early robust biomarkers for DILI.

microRNAs are small (∼22 nucleotide-long) non-protein coding RNA species involved in post-transcriptional regulation. In blood, miRNAs are stable because they are protected from degradation by extra-cellular vesicles (such as exosomes), RNA binding protein complexes (such as argonaute 2) and high-density lipoproteins. Several studies have demonstrated that hepatotoxic drugs alter miRNA levels in liver and blood, yet most of these studies have focused on acetaminophen toxicity (Wang et al., 2009; Starkey Lewis et al., 2011; Vliegenthart et al., 2015). Results have shown that acetaminophen can alter the level of ∼120 miRNAs (Vliegenthart et al., 2015), and that the liver-specific miR-122 is one of the most sensitive biomarkers in the context of acute liver injury. However, the effect of model steatotic drugs on the hepatic human miRNA profile has not been investigated yet.

In the present study, we aimed to characterize the impact of steatotic drugs on the miRNome of human hepatic cells, to find common signatures for this hepatotoxic outcome, and to investigate if these miRNA biomarkers are also altered by common MS-drugs in cultured cells. We also performed a preliminary analysis in human serum to find if these new miRNA biomarkers for steatotic drugs could have a potential application in the diagnosis of steatotic DILI, particularly in NAFLD patients, where the discrimination between iatrogenic and metabolic fat is intrinsically difficult.

## Materials and Methods

### Chemicals and Drugs

Chemical and drugs were purchased from Sigma (Madrid, Spain), Selleckchem (Madrid, Spain) and Merck (Barcelona, Spain). Steatosis-negative compounds (CITR, AMIT, and KETO), used as controls, and model steatotic drugs (VALP, DOXY, CYCA, AMIO, and TAMO) were chosen based on previous bibliography (Gomez-Lechon et al., 2007; Donato and Gomez-Lechon, 2012; Benet et al., 2014). The other chemicals used in this study were common drugs prescribed to NAFLD patients, among which we chose four antihypertensive (valsartan, IRBE, hydrochlorothiazide, and amlodipine), two antidiabetic (sitagliptin and METF), three lipid lowering drugs (FENO, SIMV, and atorvastatin), one proton-pump inhibitor (OMEP), three pain-killer (tramadol, paracetamol, and acetylsalicylic acid), two anxiolytic (LORA and diazepam) and two antidepressant (PARO and venlafaxine).

### Patients and Serum Samples

This study comprised of 44 biopsy-proven NAFLD patients. Disease severity was diagnosed according to the NASH CRN scoring system and the stage of fibrosis. Patients with other potential causes of liver disease (viral, autoimmune, metabolic such as Wilson’s disease, etc.) were excluded. Additionally, 10 non-NAFLD subjects, who underwent surgery for cholelithiasis, were included. These controls had normal liver, fasting glucose, cholesterol, TG and serum aminotransferase levels, and no evidence of viral infections. Moreover, none consumed >20 g alcohol per day. Anthropometric and analytical parameters of patients included are described in **Table 1**. Venous blood samples were collected in silicone-coated tubes and centrifuged at 2500 × *g* for 10 min. Serum samples were maintained at -80°C. This study was carried out in accordance with the ethical guidelines of the 1975 Declaration of Helsinki and with local and national laws. The Human Ethics Committee of La Fe University Hospital in Valencia approved this study (n 2013/0232), and all participants signed an informed consent.

**Table 1:** Clinical characteristics of patients studied.

	NL (*n* = 10)	NAFLD (*n* = 32)	NAFLD + Fibrate (*n* = 11)
Age (years)	38 ± 9.7	54.6 ± 10.5	54.6 ± 10.2
Sex			
Male	4 (40%)	10 (31%)	2 (18%)
Female	6 (60%)	22 (69%)	9 (82%)
Diabetes (%)	0 (0%)	14 (44%)	7 (64%)
PAH (%)	0 (0%)	15 (47%)	8 (73%)
Dyslipidemia (%)	0 (0%)	17 (53%)	11 (100%)^∗^
Metabolic Syndrome (%)	0 (0%)	19 (59%)	11 (100%)^∗^
Body mass index (kg/m^2^)	25.8 ± 3.8	30.5 ± 6.1	34.6 ± 5.41
Triglycerides (mg/dL)	102.6 ± 33.3	141.8 ± 69.3	262.5 ± 137.7^∗^
HDL-cholesterol (mg/dL)	49.1 ± 12.2	51.8 ± 9.6	44.6 ± 15.4
ALT (IU/L)	23.5 ± 17.9	56.8 ± 45.2	75.4 ± 55.9
AST (IU/L)	20.8 ± 4.1	49.2 ± 33.1	60.3 ± 38.7
γ-GT (IU/L)	37.5 ± 56.6	110.2 ± 93.3	67.4 ± 32.8
Steatosis (%)			
Grade 0	100%		
Grade 1		41.4%	0.0%
Grade 2		27.6%	30.0%
Grade 3		31.0%	70.0%^∗^
NASH (%)			
NAS_0–2	100%	27.6%	0.0%
NAS_3–4		31.0%	18.2%
NAS_5–8		41.4%	81.8%^∗^
Fibrosis (%)			
Stage 0, 1, 2	100%	72.4%	45.5%
Stage 3, 4		27.6%	54.5%
BARD score > 2	0 (0%)	13 (42%)	5 (46%)
APRI score 0.5–1	0 (0%)	10 (31%)	5 (46%)
APRI score ≥ 1.5	0 (0%)	5 (16%)	3 (27%)
NAFLD score –1.455 – 0.676	0 (0%)	10 (31%)	3 (27%)
NAFLD score > 0.676	0 (0%)	18 (56%)	8 (73%)
Drugs (%)			
ARBs	0 (0%)	6 (19%)	6 (55%)^∗^
Statins	0 (0%)	11 (34%)	4 (36%)
Metformin	0 (0%)	12 (38%)	5 (46%)
Omeprazol	0 (0%)	8 (25%)	4 (36%)
Anxiolytics	0 (0%)	8 (25%)	2 (18%)
Antidepressants	0 (0%)	4 (13%)	1 (9%)

*Data are shown as mean ± standard deviation or as number of cases (%). NL, normal liver; NAFLD, non-alcoholic fatty liver disease; HDL, high-density lipoprotein; ALT, alanine aminotransferase; AST, aspartate aminotransferase; γ-GT, gamma-glutamyltransferase; PAH, pulmonary arterial hypertension; ARBs, angiotensin II receptor blockers. ^∗^*P* < 0.05 with respect to NAFLD group.*

### Culture of HepG2 Cells, Incubation with Drugs and Cytotoxicity Assay

HepG2 cells (ATCC HB-8065) were grown in Ham’s F-12/Leibovitz L-15 (1:1, v/v) medium (Invitrogen, Barcelona), supplemented with 7% newborn calf serum, 2 mM L-glutamine, 5 mM glucose and 5 μg/mL plasmocin. HepG2 cells were seeded at 700.000 cells/3.5 cm ∅ plate.

Compound stock solutions were prepared in DMSO or water and were diluted in culture medium. HepG2 cells were exposed to drugs or solvent for 24 h. The final concentration of DMSO never exceeded 0.5% (v/v). Cytotoxicity was assessed by the mitochondrial reduction of the yellow tetrazole MTT [3-(4,5-Dimethylthiazol-2-yl)-2,5-Diphenyltetrazolium Bromide; Sigma, Madrid] to a purple formazan, in cells exposed to serially diluted drugs. Subcytotoxic concentrations (≤IC10) were calculated from the concentration-effect curves.

### Affymetrix GeneChip miRNA Arrays

Total RNA was purified from three independent HepG2 cultures treated for 24 h with CYCA (25 μM) or solvent (0.05% DMSO). miRNA expression profiles were analyzed by Affymetrix GeneChip miRNA 3.0 Arrays. Microarray hybridization and scanning were performed in IIS-LaFe microarrays platform (Hospital La Fe, Valencia, Spain). Fluorescence values were normalized with RMA algorithms (Robust Multichip Average) and DABG (Detected Above Background). Partek Genomics Suite was used in the statistical analysis applying the following parameters: PCA, ANOVA, *p*-value filtering lists and hierarchical clustering (HC) from normalized expression levels. Microarray quality control assessment and data acquisition were performed with the GeneChip^®^ Operating Software (GCOS, Affymetrix).

### Quantification of miRNAs in Cells and Serum

Total RNA was extracted from HepG2 cells with the miRNeasy Mini Kit (Qiagen, Madrid, Spain) or from 300 μL of human serum using Trizol LS reagent (Invitrogen, Barcelona, Spain) followed by the miRNeasy Mini Kit. Prior to serum RNA purification, we added 20 μg of RNase-free glycogen (Roche Applied Sciences, Barcelona, Spain) as a carrier. Purified RNA (1 μg) was reverse transcribed in two steps: polyadenylation with 1U Poly(A) polymerase from *E. coli* (New England BioLabs, Ipswich) and reverse transcription with an universal anchor primer (Supplementary Table S1) and 200U M-MLV reverse transcriptase (Invitrogen, Barcelona; Luo et al., 2012).

Diluted cDNA was amplified in a LightCycler 480 Instrument (Roche Applied Science) using LightCycler 480 Probes Master (Roche Applied Science) and the appropriate primers: a universal reverse primer, a specific forward primer for each miRNA (Supplementary Table S1) and a universal TaqMan probe (Luo et al., 2012). The concentration of miRNAs in the samples was calculated with the 2-ddC_t_ method. Sample to sample variations were normalized with the geometric mean of two miRNAs: Let-7a and miR-25, which are abundantly expressed in human cells and serum, and show low variability. Moreover, these two miRNAs showed the best stability scores in our datasets according with the NormFinder algorithm (Andersen et al., 2004).

### Quantification of Intracellular Lipids

The HCS imaging station Scan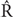 from Olympus was used to analyze neutral lipid content and MMP, by using the specific fluorescent probes BODIPY 493/503 and TMRM, respectively (Molecular Probes, Invitrogen, Barcelona; Tolosa et al., 2015).

Intracellular TG content was measured in the lipid residue using a colorimetric kit after extraction of HepG2 homogenates with a methanol-chloroform mixture (Gomez-Lechon et al., 2007).

### Data Analysis and Statistics

For microarray data analysis, PCA, ANOVA and filtering lists by *p*-value and fold-change were performed with the Partek Genomics Suite.

Heatmap and gene clustering analysis of the qRT-PCR expression data was carried out with MeV: MultiExperiment Viewer (Saeed et al., 2003). A statistical comparison of the drug-induced effects was made by hierarchical clustering and PCA using the web-based NIA Array Analysis tools (Sharov et al., 2005).

Data are expressed as mean ± SEM. The statistical significance of the mean differences was evaluated by one-way ANOVA and Tukey HSD *post hoc* test.

## Results

### Effects of Cyclosporin A (CYCA) in the miRNome of Human HepG2 Cells

**FIGURE 1:**
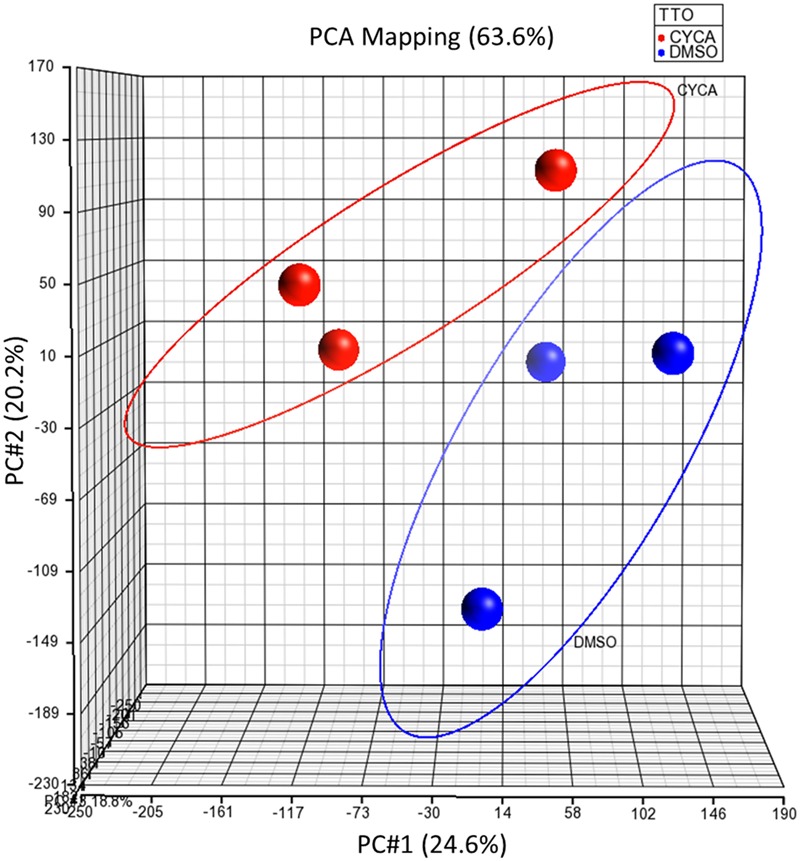
**Unsupervised PCA of global miRNA expression profiling separates CYCA treated cells from control cells.** HepG2 cultures were incubated for 24 h with CYCA (25 μM CYCA) or solvent (0.05% DMSO). miRNA expression profiles were analyzed by Affymetrix GeneChip miRNA Arrays. PCA was performed with the Partek Genomics Suite.

We investigated first the effect of a model steatotic drug, CYCA (Donato and Gomez-Lechon, 2012), on the whole miRNome of HepG2 by using Affymetrix GeneChip^®^ miRNA 3.0 Arrays. Unsupervised PCA analysis showed a separation between the DMSO and CYCA groups (**Figure 1**). Filtering results by *p*-value (<0.05) provided a list of 79 miRNAs with a significant differential expression in CYCA treated HepG2 cells. Fifty-four miRNAs were induced and 25 were repressed by CYCA (Supplementary Table S2). Among the most significantly induced miRNAs we found miR-21, -22, -23, -24, -27 and -29. We selected 35 miRNAs for further qRT-PCR validation and for investigating if this set of miRNAs were also responsive to other well-characterized steatotic drugs. The miRNA selection was based on significant differences, high confidence annotations and/or studies supporting a role in liver lipid metabolism.

### Model Steatotic Drugs Have a Common Fingerprint in the miRNA Profile of HepG2 Cells

**FIGURE 2:**
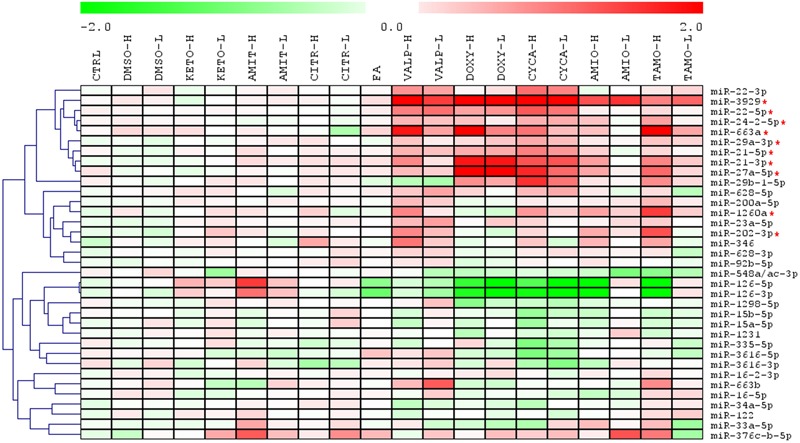
**Heatmap clustering analysis of expression patterns of 35 miRNAs reveals that steatotic drugs have a common fingerprint.** HepG2 cells were treated with model steatotic (VALP, DOXY, CYCA, AMIO, and TAMO) and non-steatotic (DMSO, KETO, AMIT, and CITR) compounds for 24 h. Two different non-cytotoxic concentrations (labeled H and L) were used. FA 0.6 mM (2:1 oleate:palmitate). Intracellular miRNA levels were analyzed by qRT-PCR-TaqMan assays in 3–5 independent experiments and normalized with the mean of miR-let7a and miR-25. Heatmap and clustering was carried out with MeV: MultiExperiment Viewer. Red asterisk denotes miRNAs upregulated by most steatotic drugs.

HepG2 cells were incubated with several steatotic and non-steatotic drugs, or with 0.6 mM FA, a condition that causes metabolic steatosis in cultured cells (Gomez-Lechon et al., 2007). The heatmap clustering analysis of 35 miRNA in **Figure 2** shows that steatotic drugs have a common fingerprint in the miRNA profile. Most of them triggered upregulation of miR-22-5p, -3929, -24-2-5p, -663a, -29a-3p, -21 (5p and 3p), -27a-5p, -1260 and -202-3p. More importantly, these miRNAs were not affected by control drugs or by FA (**Figure 2**).

Hierarchical clustering of the different drugs/conditions based on the 10 most upregulated miRNAs demonstrated that TAMO, VALP, DOXY, and CYCA cluster together (Supplementary Figure S1A). Only AMIO (both concentrations) grouped apart, though in subclusters clearly separated from the subclusters of non-steatotic compounds (KETO, AMIT, and CITR) and FA (Supplementary Figure S1A). Finally, a PCA analysis based on these 10 upregulated miRNA showed a clear separation between steatotic and non-steatotic drugs (Supplementary Figure S1B). Altogether, results indicate that steatotic drugs have a singular fingerprint in the cell miRNome, which could be useful as a predictive biosignature.

**FIGURE 3:**
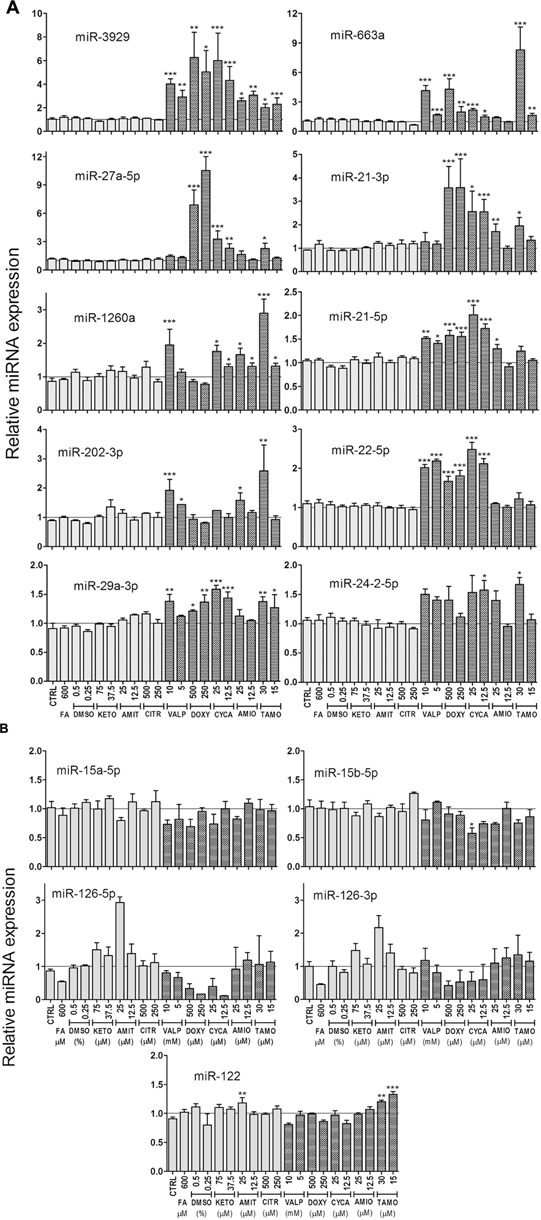
**Expression level of selected miRNAs in cells treated with steatotic and non-steatotic drugs.** Human HepG2 cells were incubated as explained in the legend to **Figure 2**. Steatotic drugs, but no FA, preferentially trigger miRNA induction **(A)**. miR-15 and miR-126 were repressed by steatotic drugs, but miR-126 were also repressed by FA **(B)**. The liver specific miR-122 did not respond to these drugs **(B)**. Bars represent the mean ± SEM (*n* = 3–5). ^∗^*p* < 0.05, ^∗∗^*p* < 0.01, ^∗∗∗^*p* < 0.001, ANOVA, Tukey *post hoc*.

A more detailed analysis of miRNA expression levels, depicted in **Figure 3A**, showed that CYCA, VALP, TAMO and DOXY induced most of the predictive miRNAs (8–9 out of 10), whereas AMIO had a significant effect in only 5–6 of these miRNAs (**Figure 3A**). This indicates that, AMIO was the most dissimilar steatosis-inducing drug. Another interesting observation is that steatotic drugs preferentially trigger miRNA induction. We attempted to validate several repressed miRNAs in microarray analysis (e.g., miR-15a-5p and miR-15b-5p) but only small negative effects were observed by qRT-PCR (20–30% repression, **Figure 3B**). Regarding miR-126, we observed that FA also downregulated this miRNA, and therefore it could not be considered a biomarker specific for drug-induced steatosis (**Figure 3B**).

Finally, none of the conditions assayed caused major effects in miR-122, a recognized marker of acute liver toxicity, in agreement with the subcytotoxic concentrations used (**Figure 3B**).

### Model Steatotic Drugs Increase the Release of miRNAs to the Culture Medium

The increase in intracellular miRNAs induced by steatotic drugs could result in increased release of miRNAs to the extracellular medium. To prove this is particularly important if these miRNAs have to be tested as circulating biomarkers. We measured the extracellular level of miRNAs (those in **Figure 3A**) after 24 h of drug-incubation and found that only steatotic drugs caused an increase in miRNA release (Supplementary Figure S2). The level of extracellular miRNAs was several orders of magnitude below the intracellular level, which did not allow a reliable analysis for the less abundant miRNAs.

### Common MS-Drugs Induce miRNA Biomarkers for Drug-Induced Steatosis

Among the frequently prescribed drugs in NAFLD patients we selected some antihypertensive, antidiabetic, lipid lowering, proton-pump inhibitor, pain-killer, anxiolytic, and antidepressant drugs, as specified in Section “Materials and Methods.” Subcytotoxic concentrations were determined from viability dose-response curves (see e.g., Supplementary Figure S3A and **Table 2**). Moreover, selected concentrations were close or below 200-fold the therapeutic peak plasma concentration (*C*_max_), which is considered a relevant dosing limit for acute drug exposure and a reasonable threshold to assess potential clinical significance (**Table 2**).

**Table 2:** Therapeutic and subcytotoxic concentrations of drugs for MS with significant effect on miRNA biomarkers for drug-induced steatosis.

Therapeutic group	Drugs	Therapeutic conc. *C*_max_ (μM)^∗^	200 ×*C*_max_ (μM)	MTT range (μM)	IC_10_ (μM)	Selected conc. (μM)
Antihypertensive	IRBE	7.7	1540	1250-10	150	150
Lipid-lowering	FENO	83.1	16620	2500-0.15	>2500	2000
	SIMV	0.02	4.0	500-3.9	7.5	7.5
Antidiabetic	METF	7.8	1560	312-0.02	>300	300
Other	OMEP	11.6	2320	5000-0.0005	500	300
	LORA	0.8	160	1250-0.02	250	250
	PARO	0.3	60	1250-0.001	125	20

*^∗^O’Brien et al. (2006).*

**FIGURE 4:**
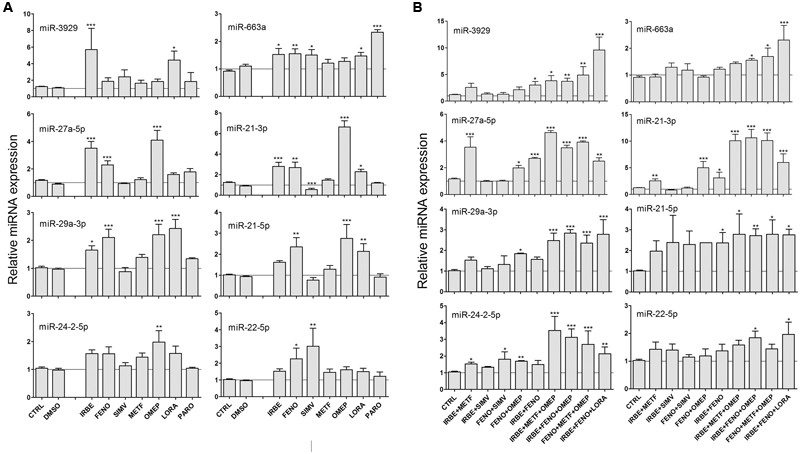
**Some common drugs for NAFLD patients induce the same miRNAs that model steatotic drugs.** HepG2 cells were treated with subcytotoxic concentrations (**Table 2**) of IRBE, FENO, SIMV, METF, OMEP, LORA, and PARO for 24 h. Drugs were added individually **(A)** or combined **(B)**. Intracellular miRNA levels were analyzed by qRT-PCR-TaqMan assays. Bars represent the mean ± SEM (*n* = 3–4). ^∗^*p* < 0.05, ^∗∗^*p* < 0.01, ^∗∗∗^*p* < 0.001, ANOVA, Tukey *post hoc*.

Many of these drugs (60%) did not have a significant effect in the miRNA biomarkers. However, we found that SIMV, METF, PARO, and particularly, IRBE, FENO, OMEP and LORA were able to significantly induce many of the miRNAs associated with drug-induced steatosis that we identified previously (**Figure 4A**).

Most of the NAFLD patients are prescribed with combined therapies and consequently we investigated the effect of some drug combinations on the miRNA profile. We found that combinations including FENO, IRBE, OMEP, LORA or METF induced all the miRNA assayed (**Figure 4B**). We also confirmed that drug combinations did not decrease cell viability by MTT assays (see e.g., Supplementary Figure S3B).

Our results show that some commonly prescribed drugs for NAFLD patients induce the same miRNA biomarkers as model steatotic drugs, and therefore, they could also induce or potentiate fatty liver in NAFLD patients.

### Common MS-Drugs Induce Lipid Accumulation in Cultured HepG2 Cells

**FIGURE 5:**
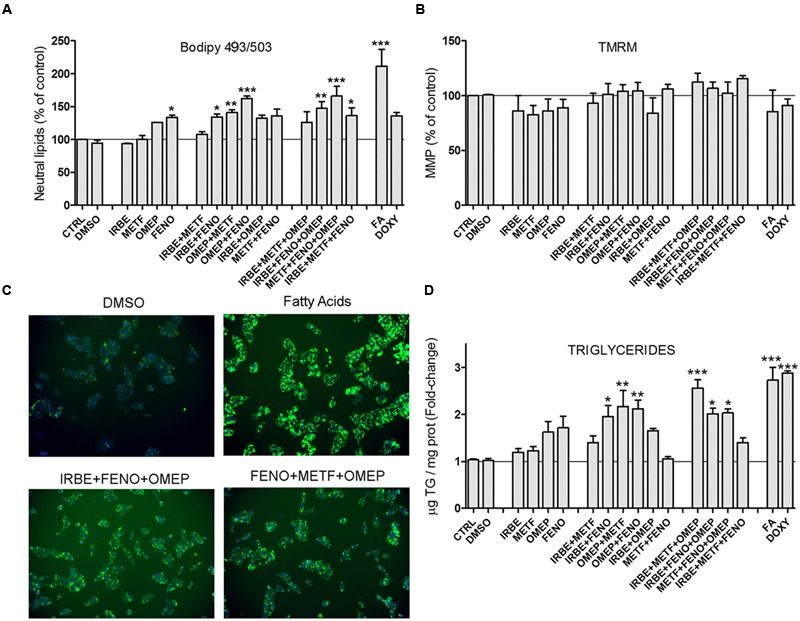
**Some common drugs for NAFLD patients induce fat accumulation in cultured human hepatic cells.** HepG2 cells were treated with IRBE, METF, OMEP, and FENO as explained in the legend to **Figure 4**. Neutral lipid content (BODIPY 493/503) **(A)** and MMP (TMRM) **(C)** were quantified by HCS. **(B)** Representative images from the HSC showing the fluorescence of BODIPY493/503 (neutral lipids; green) and Hoechst 33342 (nuclei; blue). Intracellular TG levels were also determined in parallel cultures **(D)**. Bars represent the mean ± SEM (*n* = 3–4). ^∗^*p* < 0.05, ^∗∗^*p* < 0.01, ^∗∗∗^*p* < 0.001, ANOVA, Tukey *post hoc*.

We investigated whether MS-drugs, besides inducing biomarker miRNAs were also able to induce fat accumulation in cultured human hepatic cells. HCS analysis demonstrated that BODIPY 493/503 fluorescence (neutral lipid content) increased moderately when cells were incubated with FENO, IRBE, OMEP and METF, and significantly when drugs were tested in combination (**Figures 5A,B**). Hence, in these combined settings, lipid accumulation was higher than that induced by the model steatotic drug DOXY (**Figure 5A**).

The mitochondrion is a frequent target of cytotoxic drugs. A loss in MMP can trigger energy imbalance, ROS induction, impairment in Ca^2+^ homeostasis and apoptosis. However, the drugs assayed did not affect MMP (as assessed by TMRM fluorescence; **Figure 5C**), which further supports absence of overt cytotoxicity at the selected concentrations.

Triglycerides are the major neutral lipids expected to accumulate by steatotic drugs. However, there are other neutral lipids (e.g., cholesteryl esters) that can also be stained by BODIPY 493/503. We measured TG levels enzymatically in the same experimental conditions and found results that agree well with neutral lipid levels. Once again, maximal TG accumulation was observed upon drug coincubations (**Figure 5D**). Results indicate that most of the neutral lipids accumulated by FENO, IRBE, OMEP and METF are TG.

### miRNA Biomarkers for Drug-Induced Steatosis Can be Detected in Human Serum and Are Elevated in NAFLD Patients under Fibrate Therapy

Taken into account our findings in an *in vitro* cell model, we could hypothesize that patients under combined therapies with MS-drugs, such as fibrates (FENO), ARBs (IRBE) and antidiabetics (METF), along with very frequent proton-pump inhibitors (OMEP) and/or benzodiazepines (LORA) are at risk of drug-induced steatosis. However, at present, there are not clinical biomarkers able to discriminate between iatrogenic and metabolic hepatosteatosis.

The newly *in vitro* discovered miRNAs will need future translation and validation research to prove their usefulness *in vivo*. However, as a proof-of-concept experiment, we have analyzed these biomarkers in the sera of 44 NAFLD patients and 10 non-NAFLD controls. Most of these NAFLD patients (80%) were under drug therapy for MS-associated risk factors, and only 11% of NAFLD patients did not take regular medication (**Table 1**).

The first condition that miRNA biomarkers have to fulfill is a reliable quantification in human body fluids (i.e., cycle threshold ≤36–38); because accuracy, reproducibility and precision markedly decrease when changes are detected at high cycle thresholds. We found that some of the *in vitro* miRNA biomarkers (e.g., miR-21-3p or miR-3929) were not present or below reliable limits. However, six of them (miR-21-5p, -22 (5p and 3p), -24-2-5p, -27a-5p and -29a-3p) were quantified with confidence in human serum (data not shown).

**FIGURE 6:**
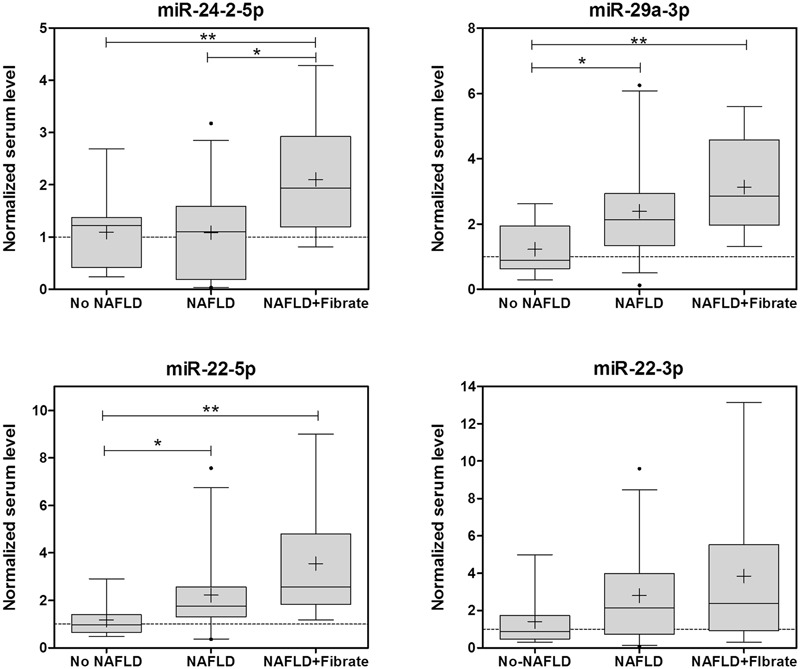
**Several biomarker miRNAs for drug-induced steatosis are induced in parallel in the sera of NAFLD patients under fibrate therapy.** The levels of miRNA biomarkers were analyzed in the serum of 44 patients with NAFLD and 10 controls. Eleven of the NAFLD patients were taken fibrates regularly. Box plots represent the 25th, the median (black line) and the 75th percentiles, a cross depicts the mean, whiskers indicate the 95th and 5th percentile and dots represent outliers. ^∗^*p* < 0.05, ^∗∗^*p* < 0.01, ANOVA, Tukey *post hoc*.

Secondly, we tested if some MS-therapies are associated with an altered serum miRNA profile in NAFLD patients. The circulating levels of these biomarkers showed no concomitant induction in patients under METF, ARBs, benzodiazepines, or OMEP (data not shown). However, patients taken fibrates showed a coincident increase in four of the six miRNAs (miR-24-2-5p, miR-22 (5p and 3p) and mR-29a-3p; **Figure 6**).

Non-alcoholic fatty liver disease patients were categorized in three histopathological groups according to the amount of fat infiltration: mild steatosis or grade 1 without an inflammatory component (G1, 6–33%), moderate steatosis or grade 2 (G2, 33–66%) and severe steatosis or grade 3 (G3, >66%; **Table 1**). Frequency distribution and chi-square test also indicated that NAFLD patients under fibrate therapy have more severe steatosis than other NAFLD patients (41% G1, 28% G2, 31% G3 in no Fibrate vs. 0% G1, 30% G2, 70% G3 in Fibrate, *p* = 0.032 Pearson, *p* = 0.08 likelihood-ratio).

The group of patients taking fibrates may also present other differential factors that could influence serum miRNAs. Comparison of clinical data in no Fibrate vs. Fibrate groups showed that only serum TG were significantly different (141.8 ± 69.3 mg/dL in no Fibrate vs. 262.5 ± 137.7 mg/dL in Fibrate, *p* = 0.017 *t*-test; **Table 1**). Thus, the differences in serum miRNAs could also be associated with serum TG. However, the correlations between serum TG and miRNA-24-2-5p (*R*_Pearson_ 0.23, *p* = 0.13), miRNA-22-5p (*R*_Pearson_ 0.20, *p* = 0.20), miRNA-22-3p (*R*_Pearson_ 0.29, *p* = 0.10) or mR-29a-3p (*R*_Pearson_ 0.058, *p* = 0.71) were no significant, whereas another non-related miRNA, miR-34a-5p, showed a strong association with serum TG (*R*_Pearson_ 0.71, *p* < 0.0001).

Results indicate that NAFLD patients under fibrate therapy have increased circulating levels of miRNA biomarkers of drug-induced steatosis, as well as more severe fatty liver. Moreover, these miRNAs are independent of serum TG levels.

## Discussion

This is the first study searching for miRNA biomarkers of drug-induced hepatic steatosis in humans, which attempts to analyze the contribution of common drugs to NAFLD. Another recent study, published while this manuscript was in preparation, searched for mechanistically linked miRNA biomarkers that distinguish between drug-induced steatosis and human NAFLD (Liu et al., 2016). However, only one of the top 10 miRNAs associated with drug-induced steatosis in that study (i.e., miR-21) was also in the list of miRNA biomarkers proposed here. The different methodological approaches of both studies could explain this lack of correlation. Liu et al. (2016) performed data mining in the open TG-GATEs (a publically available microarray database) and obtained a list of mRNAs, which were differentially expressed in the livers of rats treated with drugs; and then, inferred deregulated miRNAs by using a bioinformatics tool based on human data (miRTar). This approach assume that human and rat miRNA-mRNA interactions are 100% conserved and that all the transcriptomic alterations are mediated by miRNAs. Secondly, only one of the steatotic drugs selected by Liu et al. (2016; i.e., tetracycline) is considered a model steatotic drug, and therefore, the conclusions drawn in both studies are based on two very different sets of compounds.

The pathogenesis of NAFLD and NASH is the result of a complex interplay between host and environmental factors, and drugs are relevant environmental factors that could well be involved. Drug-induced fatty liver occurs in only some individuals, particularly those exhibiting primary risk factors (Amacher and Chalasani, 2014), and among these primary risk factors we postulate metabolic NAFLD. In this regard, non-alcoholic fatty liver is associated with mitochondrial dysfunction and diminished hepatic energy production (Wei et al., 2008), which may sensitize the liver to steatotic drugs, as many of them also have the potential to interfere with mitochondrial FA β-oxidation (Fromenty and Pessayre, 1997). The idea that NAFLD can potentiate or favor drug-induced steatosis is also supported by a recent study showing that the metabolic stress induced by a high-calorie diet in mice potentiated VPA-induced hepatosteatosis (Sathiya Priya et al., 2016).

One of the major drawbacks to investigate drug-induced steatosis in NAFLD is the inherent difficulty in differentiating the metabolic/dietary influence and the contribution of concomitant drugs. In the present study we have focused on miRNA as potential biomarkers able to discriminate between these two conditions. First, we used CYCA as a model steatotic drug and found that nearly 80 miRNAs were significantly altered in human HepG2 cells. CYCA is an immunosuppressive agent with a narrow therapeutic index because of adverse effects on different organs, including hepatotoxicity (Rezzani, 2004). CYCA induces fat accumulation in cultured hepatocytes and HepG2 cells (Donato and Gomez-Lechon, 2012). The large effect of CYCA on HepG2 miRNome is in agreement with a previous study showing that 91 miRNAs were significantly affected by 20 μM CYCA in HepG2 when combining different time-points (Van den Hof et al., 2015). However, CYCA, as many other drugs, also activates other adverse outcome pathways (e.g., cholestasis) and, therefore, not all the altered miRNAs may associate with drug-induced steatosis.

To find common signatures, we compared CYCA with other model steatotic drugs: VALP, DOXY, AMIO and TAMO. We found that several miRNAs were induced by most of these drugs, among them miR-21, -22, -24-2, -27a, -29a, -202, -663a, -1260 and -3929. It is already known that several miRNAs of this group (miR-21, -22, -24, 27a and -29a) target lipid metabolism genes and pathways (Supplementary Table S3), but there are no studies showing such an association with miR-202, -663a, -1260 and -3929. We therefore, performed mRNA target scan with all these miRNAs and looked for enriched GO terms related with lipid metabolism. This analysis shows that there is a significant enrichment in target genes with GO terms such as lipid, fatty acid, triglyceride, phospholipid, and cholesterol metabolism (biosynthesis, oxidation, transport, and storage; Supplementary Table S3). This confirms the previously reported roles of miR-21, -22, -24, 27a and -29a; and suggests novel roles for miR-202, -663a, -1260 and -3929 in lipid pathways.

The search for miRNA biomarkers was performed in human HepG2 cells, but cancer derived cell lines may have an altered miRNA profile and a disturbed response to drugs. However, previous studies have compared the miRNomes of HepG2 and hepatocytes, and found that only a 10% of miRNAs (193 out of 1881 miRNAs in miRBase) were differentially expressed in HepG2 (Bai et al., 2014). Within this list of deregulated miRNA we only found one of our miRNA biomarkers: miR-21-3p, which appears 2.8-fold upregulated in HepG2 cells. Other miRNAs with increased expression in HepG2 cells were miR-27a-3p and miR-29a-5p (2.6- and 2.9-fold, respectively; Bai et al., 2014), which are closely related to our biomarkers miR-27a-5p and miR-29a-3p. Nevertheless, most of the miRNA biomarkers investigated here were not classified as induced or repressed in HepG2 cells and, therefore, seem to have a well-preserved expression in HepG2.

Non-alcoholic fatty liver disease is strongly associated with obesity, insulin resistance, hypertension, and dyslipidemia and is regarded as the liver manifestation of the MS. Accordingly, these patients are prescribed with combined therapies for these conditions, and, therefore, are chronically exposed to a large daily number of drugs. Chronic therapies lasting several weeks or months, likely involving drug accumulation, are particularly relevant for drug-induced hepatic steatosis (Amacher and Chalasani, 2014).

We tested the possibility that MS drugs could be steatotic. We assessed the effect of 17 different common drugs of NAFLD patients in the miRNA profile and found that some of them were also able to induce the miRNAs biomarkers for drug-induced steatosis, particularly when combined. Moreover, these drugs also induced fat accumulation in HepG2 cells. The drugs with steatogenic potential were FENO, IRBE, METF, OMEP and LORA, and among them FENO was particularly significant.

Fibrates, such as FENO, are PPARα agonists widely used in clinical practice as lipid modifying agents that can improve TG plasma levels and the atherogenic lipoprotein profile. However, their effect on liver steatosis is controversial. Some studies have shown that besides lowering plasma TG, fibrates also ameliorate liver steatosis in animal models (Harano et al., 2006; Shiri-Sverdlov et al., 2006); but these studies are in clear contrast with others: serum TG were reduced but hepatic TG were increased in FENO-treated mice (Yan et al., 2014); ob/ob and LDLR-double deficient mice significantly increased the hepatic TG content and the number and size of lipid droplets after FENO (Rull et al., 2014); combined treatment of FENO with LXR or PPARγ agonists exacerbated hepatic steatosis (Gao et al., 2013; Rull et al., 2014); mice treated with FENO have even been used as a model of fatty liver (Le et al., 2014). These studies agree with the fact that FENO mediates induction of not only FA oxidation but also FA synthesis and elongation (Oosterveer et al., 2009). Our results demonstrate that both lipid accumulation and biomarker miRNAs for drug-induced steatosis are induced in HepG2 cells incubated with FENO, and that both the steatosis grade in the liver and the serum miRNA biomarkers are significantly increased in NAFLD patients under fibrate therapy. Thus, it is tempting to speculate that the use of fibrates in patients with established NAFLD may have adverse effects on the severity of the liver disease. It remains to be investigated if all the fibrates act as FENO or if some of them have no steatotic side effects, and could be more safely prescribed for NAFLD patients.

## Author Contributions

Participated in research design: ML-R, JC, MG-L, and RJ. Collected samples and conducted experiments: ML-R, IC, LT, and ÁZ. Performed data analysis and discussed the data: ML-R, IC, LT, ÁZ, and RJ. Contributed to the writing of the manuscript and to revising it critically for scientific discussions: ML-R, JC, MG-L, and RJ. All authors approved the final version to be published.

## Conflict of Interest Statement

The authors declare that the research was conducted in the absence of any commercial or financial relationships that could be construed as a potential conflict of interest.

## Abbreviations

AMIO: amiodarone
AMIT: amitriptyline
ARBs: angiotensin II receptor blockers
CITR: sodium citrate
CYCA: cyclosporin A
DILI: drug-induced liver injury
DMSO: dimethyl sulfoxide
DOXY: doxycycline
FA: fatty acids
FENO: fenofibrate
GO: gene ontology
HCS: high content screening
IRBE: irbesartan
KETO: ketotifen
LORA: lorazepam
METF: metformin
miRNA: microRNA
MMP: mitochondrial membrane potential
MS: metabolic syndrome
NAFLD: non-alcoholic fatty liver disease
NASH: non-alcoholic steatohepatitis
OMEP: omeprazole
PARO: paroxetine
PCA: principal component analysis
qRT-PCR: quantitative real-time polymerase chain reaction
SIMV: simvastatin
TAMO: tamoxifen
TG: triglycerides
TMRM: tetramethyl rhodamine methyl ester
VALP: valproate
