# Supplementary material for: New microRNA Biomarkers for Drug-Induced Steatosis and Their Potential to Predict the Contribution of Drugs to Non-alcoholic Fatty Liver Disease
Source: Front Pharmacol. 2017 Jan 25;8:3. doi: 10.3389/fphar.2017.00003 (PMC5263149; doi:10.3389/fphar.2017.00003)
Supplement: Supplementary file 1 [file Presentation_1.PDF]

Supplementary information for:

New microRNA biomarkers for drug-induced steatosis and their potential to predict the contribution of drugs to non-alcoholic fatty liver disease

Mireia López-Riera[1], Isabel Conde[1,2], Laia Tolosa[1], Ángela Zaragoza[2], José V Castell[1,3,4], M José Gómez-Lechón[1,3], Ramiro Jover[1,3,4] *

[1] Unidad de Hepatología Experimental, Instituto de Investigación Sanitaria La Fe, and

[2] Servicio Medicina Digestiva, Sección Hepatología, Hospital La Fe, Valencia, Spain.

[3] CIBERehd, ISCIII, Madrid, Spain

[4] Departamento de Bioquímica y Biología Molecular, Facultad de Medicina, Universidad de Valencia, Spain.

***Correspondence:**

Ramiro Jover

e-mail: ramiro.jover@uv.es

Supplementary Table S1: Specific forward primers for miRNA qRT-PCR

**miR-let7a**	GCAGTGAGGTAGTAGGTTGT	**miR-15a-5p**	AGCAGCACATAATGGTTTGT
**miR-25**	GCATTGCACTTGTCTCGGT	**miR-15b-5p**	ACAGCACATCATGGTTTAC
**miR-122**	ACAGTGGAGTGTGACAATG	**miR-16-5p**	GTAGCAGCACGTAAATATTG
**miR-21-5p**	AGACTAGCTTATCAGACTGATG	**miR-16-2-3p**	CCAATATTACTGTGCTGCTT
**miR-21-3p**	AACACCAGTCGATGGGCTGT	**miR-92b-5p**	AGGGACGGGACGCGGTGCA
**miR-22-3p**	GGCTGCCAGTTGAAGAACT	**miR-126-5p**	GCATTATTACTTTTGGTACG
**miR-22-5p**	GAGTTCTTCAGTGGCAAGC	**miR-126-3p**	TCGTACCGTGAGTAATAATG
**miR-23a-5p**	GGGTTCCTGGGGATGGGAT	**miR-200a-5p**	CATCTTACCGGACAGTGCTG
**miR-24-2-5p**	GCCTACTGAGCTGAAACACA	**miR-202-3p**	AAGGTATAGGGCATGGGA
**miR-27a-5p**	AGGGCTTAGCTGCTTGTGAG	**miR-335-5p**	CAAGAGCAATAACGAAAAATG
**miR-29a-3p**	GCAGTAGCACCATCTGAAATC	**miR-346**	TGCCCGCATGCCTGCCTCT
**miR-29b-1-5p**	CTGGTTTCATATGGTGGTTTAG	**miR-376c/b-5p**	GGTGGATATTCCTTCTATGT
**miR-33a-5p**	GCCAGGTGCATTGTAGTTG	**miR-1298-5p**	ATTCGGCTGTCCAGATGT
**miR-34a-5p**	TCAGTGGCAGTGTCTTAGCT	**miR-3613-5p**	GTTGTACTTTTTTTTTTGTTC
**miR-548a/ac-3p**	GAAAACTGGCAATTACTTTTG	**miR-3613-3p**	ACAAAAAAAAAAGCCCAACC
**miR-628-5p**	ATGCTGACATATTTACTAGAG	**miR-3929**	GGCTGATGTGAGTAGACCACT
**miR-628-3p**	GTCTAGTAAGAGTGGCAGT	**miR-1231**	GTGTCTGGGCGGACAGCT
**miR-663b**	TACCCGGCCGTGCCTGA	**miR-1260a**	ATCCCACCTCTGCCACCA
**miR-663a**	AGGGGCGCCGCGGGAC		

Universal RT anchor primer: CGACTCGATCCAGTCTCAGGGTCCGAGGTATTCGATCC TAACCCTCTCCTCGGTATCGAGTCGCACTTTTTTTTTTTTVN

Universal PCR reverse primer: CCAGTCTCAGGGTCCGAGGTATTC

Universal TaqMan probe: FAM-TCTCCTCGGTATCGAGTCGCACT-TAMRA

Supplementary Table S2: miRNAs with altered expression in HepG2 cells treated with CYCA. Affymetrix GeneChip® miRNA 3.0 Arrays

	Transcript ID	p-value (CYCA vs. DMSO)	Ratio (CYCA vs. DMSO)
**Significantly induced**			
1	**hsa-miR-27a-5p**	0.003	14.62
2	hsa-miR-4417	0.02	9.27
3	**hsa-miR-23a-5p**	0.003	6.39
4	**hsa-miR-1231**	0.005	5.85
5	**hsa-miR-200a-5p**	0.04	5.05
6	**hsa-miR-92b-5p**	0.05	5.04
7	**hsa-miR-29b-1-5p**	0.00003	4.69
8	**hsa-miR-21-3p**	0.002	4.38
9	**hsa-miR-22-5p**	0.004	3.72
10	**hsa-miR-1260a**	0.002	3.48
11	**hsa-miR-3929**	0.002	3.28
12	**hsa-miR-346**	0.05	3.11
13	**hsa-mir-21**	0.03	3.07
14	hsa-miR-3195	0.02	3.07
15	hsa-miR-4443	0.0004	2.71
16	hsa-miR-6768-5p	0.04	2.52
17	**hsa-mir-663a**	0.03	2.49
18	hsa-miR-4428	0.04	2.42
19	hsa-miR-1343-5p	0.01	2.38
20	hsa-miR-4721	0.01	2.37
21	**hsa-miR-628-3p**	0.08	2.34
22	hsa-miR-4497	0.01	2.34
23	**hsa-mir-663b**	0.02	2.33
24	**hsa-mir-663b**	0.01	2.31
25	hsa-miR-6836-5p	0.02	2.16
26	hsa-mir-6746	0.04	2.11
27	hsa-miR-3178	0.002	2.01
28	hsa-miR-181b-5p	0.04	2.00
29	**hsa-miR-24-2-5p**	0.002	1.98
30	hsa-miR-148a-5p	0.04	1.97
31	hsa-miR-4299	0.02	1.91
32	hsa-mir-4767	0.05	1.91
33	hsa-mir-3180-1	0.001	1.84
34	hsa-mir-3180-2	0.001	1.84
35	hsa-mir-3180-3	0.001	1.84
36	**hsa-miR-27a-3p**	0.02	1.80
37	hsa-miR-4749-5p	0.03	1.75
38	hsa-miR-4783-3p	0.04	1.75
39	**hsa-miR-22-3p**	0.0002	1.75
40	hsa-miR-5100	0.02	1.74
41	hsa-miR-92a-1-5p	0.02	1.73
42	hsa-mir-5095	0.01	1.70
43	hsa-miR-4786-3p	0.01	1.70
44	hsa-miR-4758-5p	0.02	1.70
45	hsa-miR-486-3p	0.04	1.67
46	hsa-mir-4734	0.03	1.64
47	hsa-miR-942-3p	0.04	1.62
48	hsa-miR-550a-3-5p	0.04	1.61
49	hsa-miR-3651	0.03	1.61
50	hsa-mir-658	0.03	1.57
51	hsa-miR-23a-3p	0.002	1.57
52	hsa-miR-615-5p	0.01	1.56
53	hsa-miR-6884-3p	0.03	1.52
54	**hsa-miR-29a-3p**	0.05	1.31

	Transcript ID	p-value (CYCA vs. DMSO)	Ratio (CYCA vs. DMSO)
**Significantly repressed**			
1	hsa-miR-1257	0.02	0.67
2	**hsa-miR-16-5p**	0.04	0.64
3	hsa-mir-550a-1	0.02	0.63
4	hsa-mir-550a-2	0.02	0.63
5	hsa-mir-550a-3	0.02	0.63
6	hsa-miR-18b-5p	0.01	0.63
7	hsa-let-7g-5p	0.01	0.63
8	hsa-mir-8084	0.03	0.62
9	hsa-miR-6777-5p	0.02	0.61
10	hsa-miR-6778-5p	0.01	0.60
11	hsa-mir-4521	0.03	0.60
12	hsa-miR-551b-5p	0.05	0.59
13	**hsa-miR-15a-5p**	0.04	0.56
14	hsa-miR-4521	0.03	0.53
15	hsa-miR-6880-5p	0.03	0.51
16	hsa-miR-6740-5p	0.04	0.49
17	hsa-miR-6779-5p	0.005	0.49
18	**hsa-miR-15b-5p**	0.01	0.49
19	hsa-miR-4732-5p	0.01	0.48
20	hsa-miR-3149	0.001	0.45
21	hsa-miR-6511b-5p	0.004	0.41
22	hsa-miR-6769a-5p	0.01	0.41
23	hsa-miR-1244	0.03	0.14
24	**hsa-miR-628-5p**	0.001	0.13
25	**hsa-miR-1298-3p**	0.02	0.05
**Repressed (based only in low ratio)**			
	**hsa-miR-202-3p**	0.11	**0.53**
	**hsa-miR-126-3p**	0.39	**0.43**
	**hsa-miR-126-5p**	0.38	**0.35**
	**hsa-miR-376c-5p**	0.33	**0.22**
	**hsa-miR-16-2-3p**	0.25	**0.13**
	**hsa-miR-335-5p**	0.08	**0.10**
	**hsa-miR-548a-3p**	0.14	**0.06**
**Other miRNAs selected: important roles in liver lipid metabolism**			
	**hsa-miR-21-5p**	0.70	1.10
	**hsa-miR-122-5p**	0.23	0.86
	**hsa-miR-33a-5p**	0.30	0.82
	**hsa-miR-34a-5p**	0.24	0.80

miRNAs selected for validation by qRT-PCR TaqMan assays are highlighted in bold

Supplementary Table S3A: Target genes related to lipid metabolism of several biomarker miRNAs for drug-induced steatosis.

miRNA	Target genes related to lipid metabolism	References
**miR-21**	FABP7, PPARα, IGFBP3	[1]
**miR-22**	ACLY, ELOVL6, MAT1A, PTEN	[2-4]
**miR-24**	FASN, SREBP1c, SCD, ACAC, INSIG1, ARNT	[5-7]
**miRNA-27a**	LDLR, RXRα, PPARγ, PPARα, FASN, SREBPs, ApoA1, ApoB, ApoE3	[8-11]
**miR-29a**	LPL, PTEN, FXR, SIRT1, AHR	[12-15]

[1] Yang Z, Cappello T, Wang L. Emerging role of microRNAs in lipid metabolism. Acta pharmaceutica Sinica B 2015;5:145-150.

[2] Koufaris C, Valbuena GN, Pomyen Y, Tredwell GD, Nevedomskaya E, Lau CH, et al. Systematic integration of molecular profiles identifies miR-22 as a regulator of lipid and folate metabolism in breast cancer cells. Oncogene 2015.

[3] Koturbash I, Melnyk S, James SJ, Beland FA, Pogribny IP. Role of epigenetic and miR-22 and miR-29b alterations in the downregulation of Mat1a and Mthfr genes in early preneoplastic livers in rats induced by 2-acetylaminofluorene. Molecular carcinogenesis 2013;52:318-327.

[4] Bar N, Dikstein R. miR-22 forms a regulatory loop in PTEN/AKT pathway and modulates signaling kinetics. PloS one 2010;5:e10859.

[5] Wang H, Luo J, Chen Z, Cao WT, Xu HF, Gou DM, et al. MicroRNA-24 can control triacylglycerol synthesis in goat mammary epithelial cells by targeting the fatty acid synthase gene. Journal of dairy science 2015;98:9001-9014.

[6] Ng R, Wu H, Xiao H, Chen X, Willenbring H, Steer CJ, et al. Inhibition of microRNA-24 expression in liver prevents hepatic lipid accumulation and hyperlipidemia. Hepatology (Baltimore, Md) 2014;60:554-564.

[7] Oda Y, Nakajima M, Mohri T, Takamiya M, Aoki Y, Fukami T, et al. Aryl hydrocarbon receptor nuclear translocator in human liver is regulated by miR-24. Toxicology and applied pharmacology 2012;260:222-231.

[8] Alvarez ML, Khosroheidari M, Eddy E, Done SC. MicroRNA-27a decreases the level and efficiency of the LDL receptor and contributes to the dysregulation of cholesterol homeostasis. Atherosclerosis 2015;242:595-604.

[9] Ji J, Zhang J, Huang G, Qian J, Wang X, Mei S. Over-expressed microRNA-27a and 27b influence fat accumulation and cell proliferation during rat hepatic stellate cell activation. FEBS letters 2009;583:759-766.

[10] Li S, Li J, Fei BY, Shao D, Pan Y, Mo ZH, et al. MiR-27a promotes hepatocellular carcinoma cell proliferation through suppression of its target gene peroxisome proliferator-activated receptor gamma. Chinese medical journal 2015;128:941-947.

[11] Shirasaki T, Honda M, Shimakami T, Horii R, Yamashita T, Sakai Y, et al. MicroRNA-27a regulates lipid metabolism and inhibits hepatitis C virus replication in human hepatoma cells. Journal of virology 2013;87:5270-5286.

[12] Mattis AN, Song G, Hitchner K, Kim RY, Lee AY, Sharma AD, et al. A screen in mice uncovers repression of lipoprotein lipase by microRNA-29a as a mechanism for lipid distribution away from the liver. Hepatology (Baltimore, Md) 2015;61:141-152.

[13] Kong G, Zhang J, Zhang S, Shan C, Ye L, Zhang X. Upregulated microRNA-29a by hepatitis B virus X protein enhances hepatoma cell migration by targeting PTEN in cell culture model. PloS one 2011;6:e19518.
[14] Li J, Zhang Y, Kuruba R, Gao X, Gandhi CR, Xie W, et al. Roles of microRNA-29a in the antifibrotic effect of farnesoid X receptor in hepatic stellate cells. Molecular pharmacology 2011;80:191-200.
[15] Kurtz CL, Fannin EE, Toth CL, Pearson DS, Vickers KC, Sethupathy P. Inhibition of miR-29 has a significant lipid-lowering benefit through suppression of lipogenic programs in liver. Scientific reports 2015;5:12911.

Supplementary Table S3B: Enriched GO terms in target genes of miRNA induced by steatotic drugs.

PathName - GOBP name**	miRNAs	p-value range	p-value range (BH-corrected)*
**Cellular lipid metabolic process**	miR-21-5p; -22-5p; -24-2-5p; -27a-5p; -202-3p; -663a; -3929	0.000-0.038	0.000-0.046
**Lipid biosynthetic process**	miR-21-5p; -24-2-5p; -27a-5p; -29a-3p; -202-3p; -663a; -3929	0.004-0.039	0.018-0.047
**Lipid transport**<br>**Lipoprotein transport**<br>**Low density lipoprotein particle clearance**	miR-21-5p; -27a-5p; -29a-3p	0.011-0.045	0.029-0.042
**Unsaturated fatty acid metabolic process**<br>**Fatty acid metabolic process**<br>**Fatty acid homeostasis**	miR-21 (3p-5p); -22-5p; 24-2-5p; -27a-5p; -29a-3p; -202-3p; -663a; -1260a; -3929	0.001-0.046	0.009-0.049
**Regulation of fatty acid biosynthetic process**<br>**Long chain fatty acyl CoA biosynthetic process**<br>**Fatty acid biosynthetic process**<br>**Positive regulation of fatty acid biosynthetic process**<br>**Triglyceride biosynthetic process**	miR-21-5p; -22-5p; 24-2-5p; -27a-5p; -663a; -1260a;	0.000-0.047	0.003-0.049
**Positive regulation of fatty acid beta oxidation**<br>**Regulation of fatty acid oxidation**<br>**Fatty acid beta oxidation**<br>**Fatty acid beta oxidation using acyl CoA dehydrogenase**<br>**Fatty acid beta oxidation using acyl CoA oxidase**<br>**Lipid catabolic process**	miR-21-5p; -22-5p; 24-2-5p; -27a-5p; 29a-3p; -202-3p; -663a; -1260a; -3929	0.000-0.042	0.003-0.049

**Negative regulation of lipid storage**<br>**Negative regulation of cholesterol storage**	miR-21-5p and -22-5p	0.004-0.045	0.021-0.049
**Cellular response to cholesterol**<br>**Response to cholesterol**<br>**Cholesterol biosynthetic process**<br>**Intracellular cholesterol transport**<br>**Positive regulation of cholesterol efflux**<br>**Cholesterol homeostasis**	miR-21-5p; -22-5p; -27a-5p; -663a; -1260a	0.005-0.042	0.021-0.049
**Glycerophospholipid biosynthetic process**<br>**Phospholipid biosynthetic process**<br>**Phospholipid homeostasis**	miR-21-3p; -22-5p; -24-2-5p; -27a-5p; -29a-3p; -202-3p; -663a; -1260a; -3929	0.000-0.047	0.002-0.049
**Phospholipid metabolic process**	miR-21-5p; -22-5p; -24-2-5p; -27a-5p; -29a-3p; -202-3p; -663a; -1260a; -3929	0.000-0.005	0.000-0.019
**Phospholipid translocation**<br>**Phospholipid transport**	miR-21 (3p-5p); -22-5p; -27a-5p; -202-3p; -1260a; -3929	0.001-0.044	0.008-0.044
**Positive regulation of fat cell differentiation**<br>**Negative regulation of fat cell differentiation**<br>**Fat cell differentiation**	miR-21 (3p-5p); -22-5p; -27a-5p; -29a-3p; -202-3p; -663a; -1260a; -3929	0.002-0.047	0.012-0.045
**Energy reserve metabolic process**<br>**Metabolic process**	miR-21-3p; -22-5p; -24-2-5p; -27a-5p; -29a-3p; -202-3p; -1260a	0.004-0.040	0.018-0.049

The search was conducted with the web-based application miRWalk 2.0. Parameters were customized to search only in the 3’UTR region and accept a minimum seed length of 7 bp.

**GOBP: over-represented GO Biological Process.

*The Benjamini–Hochberg (BH) procedure controls the false discovery rate (at level alpha).

Figure S1A

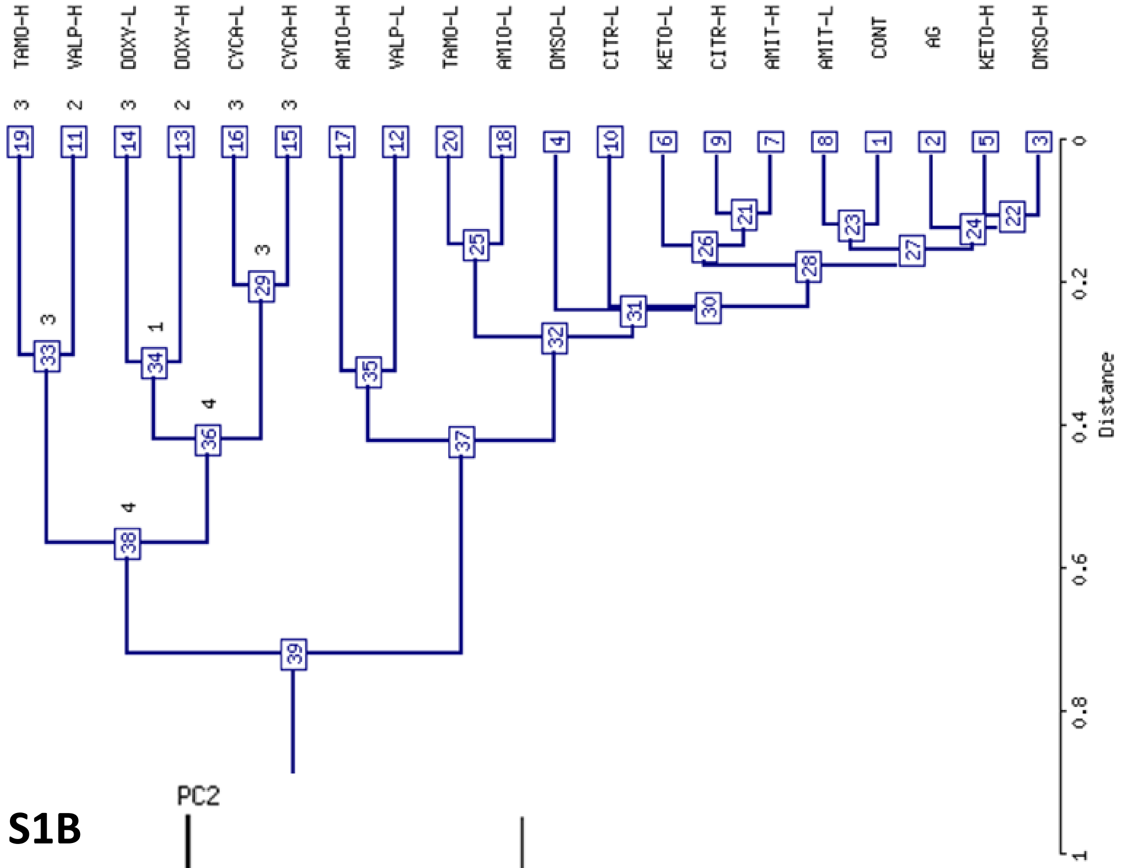

Figure S1B

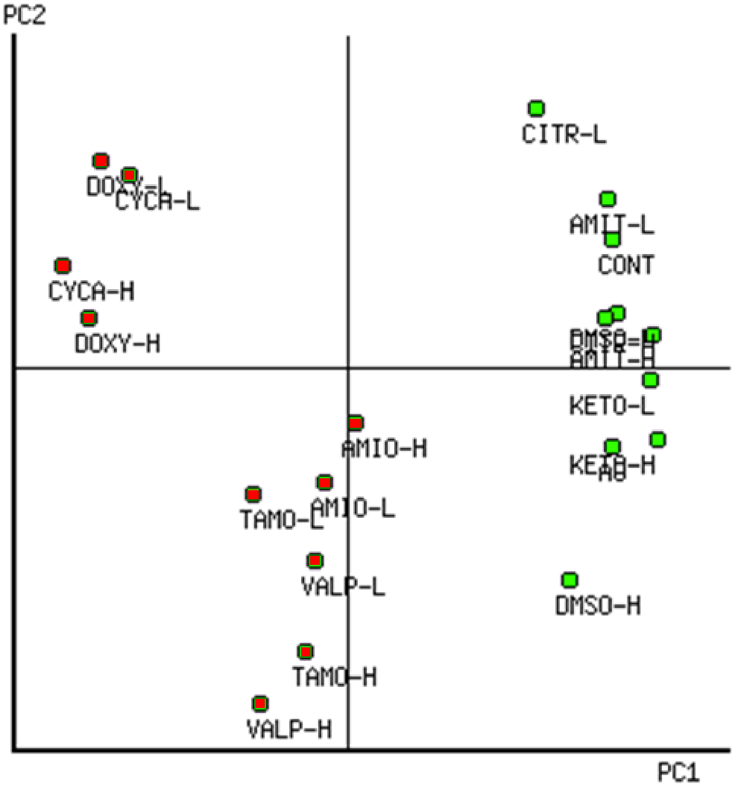

Supplementary Fig. S1: Hierarchical clustering analysis (A) and PCA (B) of the different treatment / conditions based on the expression of 10 miRNAs biomarkers of drug-induced steatosis (H: higher conc. L: Lower conc. AG: 0.6 mM fatty acids)

Figure S2

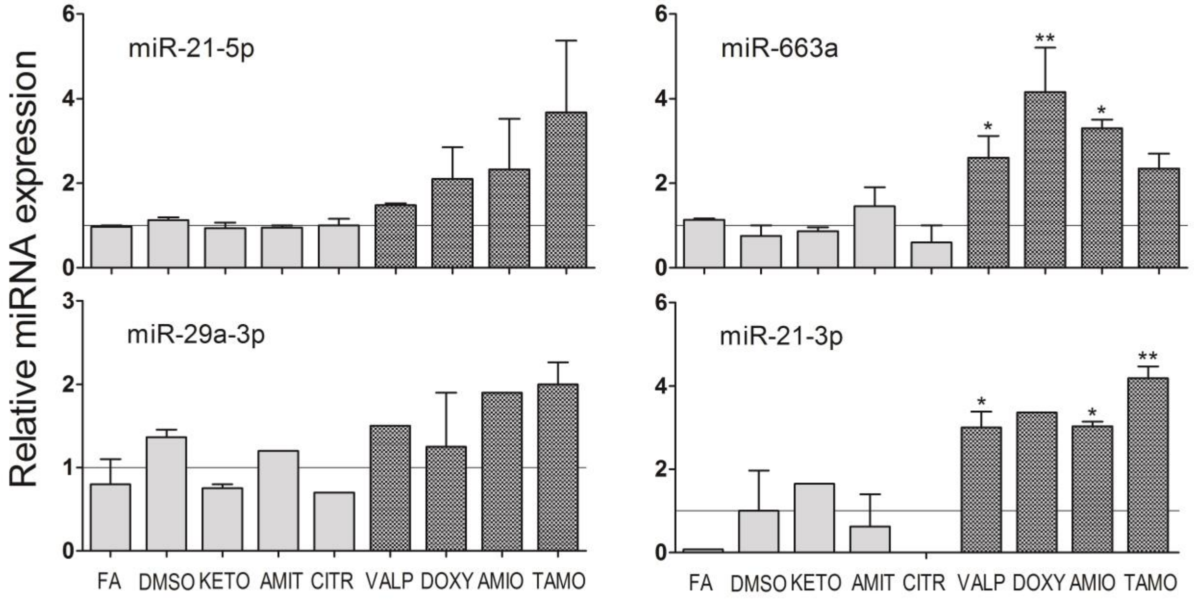

Supplementary Fig. S2: Release of miRNA biomarkers to culture medium by HepG2. Human HepG2 cells were incubated with model steatotic (10 mM VALP, 250 µM DOXY, 25 µM AMIO and 30 µM TAMO) and non-steatotic (0.25% DMSO, 75 µM KETO, 25µM AMIT and 500 µM CITR) compounds. FA: fatty acids 0.6 mM (2:1 oleate:palmitate). Culture medium was collected at t=0h and t=24h and centrifuged at 7500xg for 10min. Extracellular RNA was purified from 300µL of medium and specific miRNAs were quantified and normalized as described in Materials and Methods. Bars represent the mean ± SEM. * $p<0.05$, ** $p<0.01$.

Figure S3A

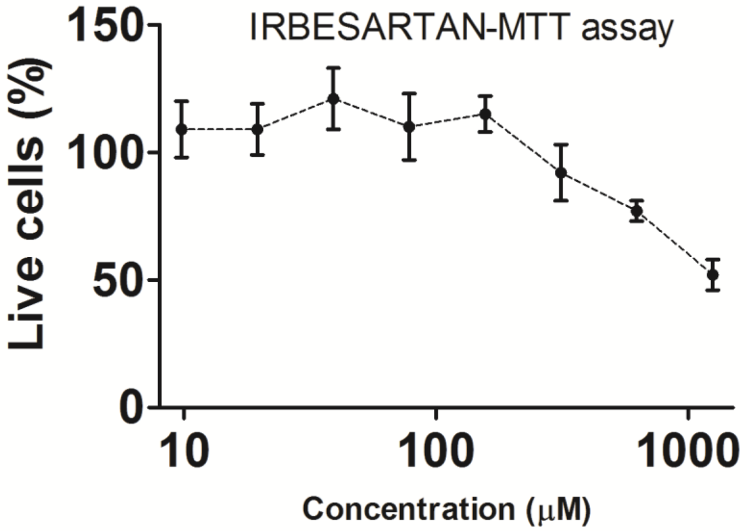

Figure S3B

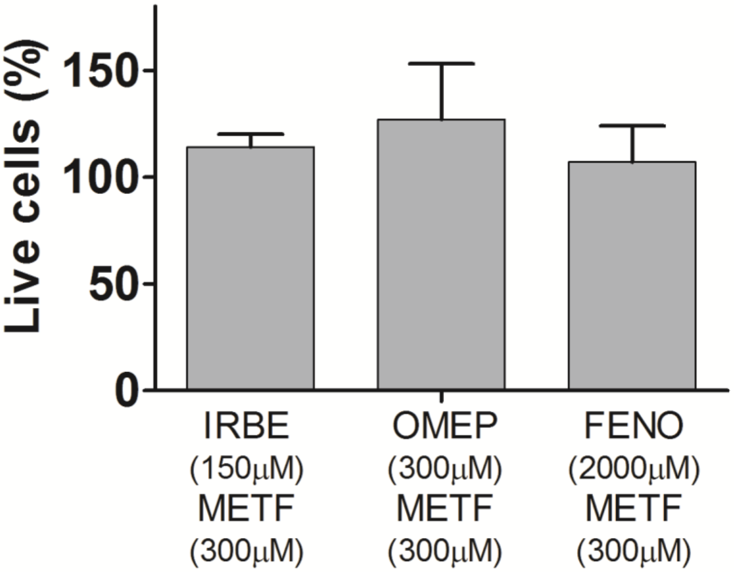

Supplementary Fig. S3: Cytotoxicity of some MS drugs in HepG2 cells by MTT assays. Cells were incubated with increasing concentrations of IRBE (**A**), or coincubated with 2 drugs at selected concentrations (**B**).
